# O Risco de Doença Cardiovascular Segundo o Escore Não Laboratorial da OMS em uma População Brasileira Selecionada: Percentis da Distribuição e Concordância com o Escore Laboratorial

**DOI:** 10.36660/abc.20240002

**Published:** 2024-07-31

**Authors:** Fernando Yue Cesena, Giuliano Generoso, Itamar de S. Santos, Alexandre C. Pereira, Marcio S. Bittencourt, Raul D. Santos, Paulo A. Lotufo, Isabela M. Benseñor

**Affiliations:** 1 Instituto Dante Pazzanese de Cardiologia São Paulo SP Brasil Instituto Dante Pazzanese de Cardiologia, São Paulo, SP – Brasil; 2 Centro de Pesquisa Clínica e Epidemiológica Hospital Universitário Universidade de São Paulo São Paulo SP Brasil Centro de Pesquisa Clínica e Epidemiológica, Hospital Universitário, Universidade de São Paulo, São Paulo, SP – Brasil; 3 Genetics Department Harvard Medical School Boston Massachusetts EUA Genetics Department, Harvard Medical School, Boston, Massachusetts – EUA; 4 Heart and Vascular Institute University of Pittsburgh Medical Center Pittsburgh Pennsylvania EUA Heart and Vascular Institute, University of Pittsburgh Medical Center, Pittsburgh, Pennsylvania – EUA; 5 Instituto do Coração Hospital das Clínicas da Faculdade de Medicina Universidade de São Paulo São Paulo SP Brasil Instituto do Coração (InCor), Hospital das Clínicas da Faculdade de Medicina da Universidade de São Paulo, São Paulo, SP – Brasil; 6 Hospital Israelita Albert Einstein São Paulo SP Brasil Hospital Israelita Albert Einstein, São Paulo, SP – Brasil

**Keywords:** Doenças Cardiovasculares, Fatores de Risco de Doenças Cardíacas, Índice de Massa Corporal, Medição de Risco, Organização Mundial da Saúde

## Introdução

Em 2019, a Organização Mundial da Saúde (OMS) publicou gráficos revisados para estimar o risco de doenças cardiovasculares (DCV) em 10 anos.^1^ Foram propostos dois modelos: um baseado em exames laboratoriais incluindo colesterol total plasmático e presença ou ausência de diabetes mellitus como preditores, e outro baseado no índice de massa corporal (IMC). Em locais onde recursos são escassos e em situações de consultório em que os níveis de colesterol e informações sobre diabetes não estão disponíveis, o escore baseado no IMC pode ser usado.^2^

Em um estudo anterior, determinamos percentis da distribuição do risco de DCV da OMS laboratorial, de acordo com sexo e idade, na linha de base da população do Estudo Longitudinal de Saúde do Adulto (ELSA-Brasil).^3^ Esses percentis foram associados ao risco calculado de DCV aterosclerótica até 75 anos, independentemente do risco estimado em 10 anos. A expressão do percentil de risco tem sido proposta para aumentar a conscientização do risco e melhorar a adesão às medidas preventivas.^3-5^

Neste estudo, buscamos (1) determinar percentis específicos para sexo e idade da distribuição do risco de DCV pelo escore não laboratorial da OMS na população brasileira e (2) avaliar a concordância entre os escores de risco de DCV laboratorial e não laboratorial da OMS.

## Método

Este estudo é uma análise transversal dos dados basais do ELSA-Brasil, coletados no período de 2008 a 2010. O ELSA-Brasil é uma coorte prospectiva de 15.105 funcionários de etnias mistas de universidades públicas e instituições de pesquisa em seis cidades brasileiras.^6,7^ Foram incluídos participantes de 40 a 74 anos e excluídos aqueles que apresentavam infarto do miocárdio, acidente vascular encefálico ou procedimentos de revascularização prévios. O protocolo do ELSA-Brasil foi aprovado pelo comitê de ética de cada instituição participante e todos os participantes deram consentimento informado por escrito.

A hipertensão foi considerada como pressão arterial sistólica ≥140 mmHg, pressão arterial diastólica ≥90 mmHg ou uso de medicação para controle da pressão arterial. Diabetes mellitus foi definida como diagnóstico autorreferido, uso de medicação específica, glicemia em jejum ≥126 mg/dL, hemoglobina glicada ≥6,5% ou glicemia de 2 horas ≥200 mg/dL após carga de 75 g em teste oral de tolerância à glicose. Definições detalhadas das demais variáveis relatadas neste estudo podem ser encontradas em outras publicações.^3^ Os níveis plasmáticos de colesterol total e o IMC foram categorizados de acordo com os grupos usados no cálculo do risco de DCV da OMS.^1,2^

O risco de um primeiro evento de DCV fatal ou não fatal em 10 anos (relacionado a doença coronariana ou acidente vascular encefálico) foi calculado usando os escores de risco de DCV da OMS atualizados em 2019, calibrados para a América Latina Tropical.^1,2^

Para estabelecer percentis da distribuição do escore de risco de DCV não laboratorial da OMS, primeiramente ordenamos todos os valores possíveis do risco calculado dentro de cada faixa etária. Em seguida, determinamos o percentil de distribuição correspondente a cada escore. Foram realizadas análises separadas para cada sexo.

O teste do sinal foi aplicado para comparar os escores de risco, uma vez que as diferenças entre as observações pareadas não eram simétricas. Um valor de p < 0,05 foi considerado estatisticamente significativo. A concordância entre os valores de risco foi avaliada pelos diagramas de Bland-Altman. Todas as análises foram realizadas utilizando o software R e Microsoft Excel. O pacote do R Shiny foi utilizado para desenvolver uma aplicação web para cálculo do risco de DCV em 10 anos e o percentil correspondente para sexo e idade.

## Resultados

A população do estudo (n = 13.366) foi caracterizada por maior presença feminina (55%) e idade mediana (intervalo interquartil [IQR]) de 52 (46, 59) anos (Figura Complementar 1, Tabela Complementar 1).

**Figura 1:**
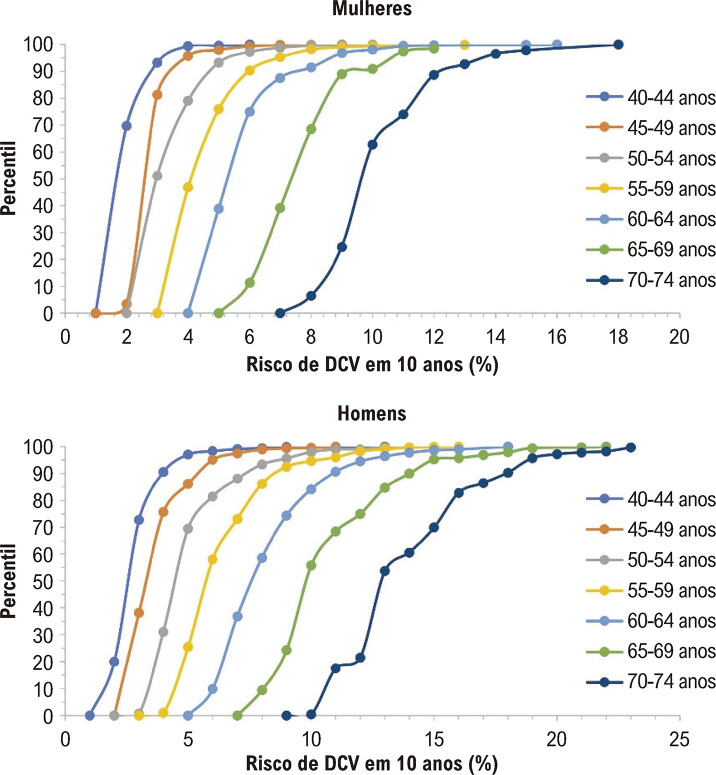
– Percentis da distribuição do risco predito de doença cardiovascular (DCV) em 10 anos, de acordo com sexo e faixa etária. O risco de DCV foi calculado pelo escore de risco não laboratorial da Organização Mundial da Saúde.

A Figura Complementar 2 mostra a distribuição do risco predito de DCV em 10 anos de acordo com sexo e faixa etária, enquanto as Tabelas Complementares de 2 a 8 apresentam os percentis dessa distribuição. Esses percentis permitiram o desenvolvimento de gráficos de risco versus percentil, de acordo com sexo e faixa etária (Figura 1).

**Figura 2:**
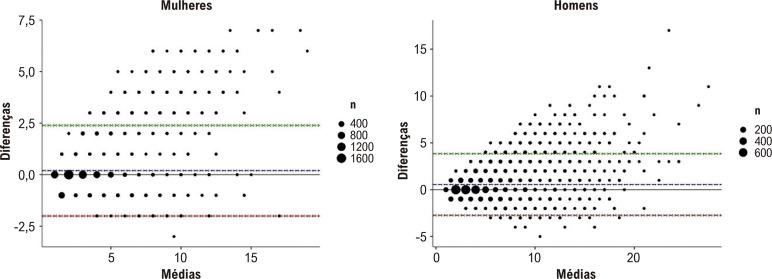
– Diagramas de Bland-Altman para a concordância entre os escores de risco de DCV laboratorial e não laboratorial da OMS, de acordo com o sexo. A linha azul representa a média de todas as diferenças (escores laboratoriais menos escores não laboratoriais), enquanto as linhas vermelha e verde representam os limites de concordância de 95% inferior e superior, respectivamente. DCV: doença cardiovascular.

Na amostra total, o risco baseado no IMC foi ligeiramente inferior ao risco baseado em exames laboratoriais (mediana [IQR] 3% [2%, 5%] versus 4% [2%, 6%], respectivamente, p <0,001). A Figura 2 mostra a concordância entre os escores de risco laboratorial e não laboratorial em mulheres e homens. Os escores coincidiram na maioria dos participantes (7.884 [59%]). A diferença entre os valores (risco laboratorial menos risco não laboratorial) variou entre -3% e 7% para as mulheres e entre -5% e 17% para os homens.

As Figuras Complementares 3 a 5 mostram a concordância entre escores de risco laboratorial e não laboratorial, de acordo com subgrupos de interesse. Entre os indivíduos sem diabetes mellitus, os valores foram iguais em 7.873 (70,5%), enquanto o risco laboratorial foi numericamente superior ao risco baseado no IMC em 2.176 (99,5%) participantes com diabetes. Quanto maior o nível de colesterol total e menor o IMC, maior foi a tendência para um risco laboratorial mais elevado em comparação com o risco não laboratorial.

Uma aplicação web para calcular riscos de DCV e percentis por sexo e idade pode ser acessada em https://bit.ly/3sGsIgK. Um código R para criar novas variáveis para o risco de DCV da OMS baseado em dados laboratoriais ou no IMC em um conjunto de dados, além dos percentis correspondentes para sexo e idade, está disponível em https://bit.ly/3Pov250.

## Discussão

A avaliação do risco de DCV com base no IMC é uma opção quando exames de colesterol e glicemia não estão disponíveis,^2^ podendo ser um método mais simples para os membros da comunidade calcularem o seu próprio risco. Nossos achados indicam que o risco baseado no IMC coincide com o risco laboratorial na maioria das circunstâncias, mas pode ser substancialmente menor na presença de diabetes mellitus e colesterol total muito elevado. Pode-se fazer a hipótese de que o risco não baseado em laboratório subestima o risco real nestes subgrupos de alto risco. No entanto, conclusões definitivas não podem ser feitas, uma vez que métricas de desempenho dos escores não foram avaliadas. Observamos também que o risco baseado no IMC tende a ser menor do que o escore laboratorial à medida que o IMC diminui. O escore com melhor precisão entre indivíduos com baixo IMC permanece uma questão em aberto.

As limitações deste estudo incluem o fato de a população estudada não ser representativa da população brasileira, com maior proporção feminina, mais brancos e menos pardos em comparação com o Censo Brasileiro de 2022.^8^ Além disso, esta análise de concordância pode não ser aplicada a outras populações com características demográficas diferentes. Por fim, deve-se ressaltar que o cálculo do risco representa apenas o primeiro passo na estratificação do risco de DCV, e a prática clínica também deve considerar diversos outros fatores e avaliação da aterosclerose subclínica.^9^

Para concluir, este estudo estabeleceu percentis da distribuição do risco de DCV da OMS não baseado em laboratório por sexo e idade na população brasileira. Em comparação com a estratégia baseada em laboratório, a abordagem não laboratorial leva ao mesmo escore de risco na maioria dos indivíduos, mas tende a subestimar o risco em homens, indivíduos com diabetes, LDL-c mais elevado ou IMC mais baixo.
