# Supplementary material for: O Risco de Doença Cardiovascular Segundo o Escore Não Laboratorial da OMS em uma População Brasileira Selecionada: Percentis da Distribuição e Concordância com o Escore Laboratorial
Source: Arq Bras Cardiol. 2024 Jul 31;121(7):e20240002. [Article in Portuguese] doi: 10.36660/abc.20240002 (PMC11464092; doi:10.36660/abc.20240002)
Supplement: Translation

# Translation: The WHO Cardiovascular Disease Risk (Non-Laboratory-Based) in a Selected Brazilian Population: Percentiles of Distribution And Agreement With Laboratory-Based Scores

**DOI:** 10.36660/abc.20240002i

**Authors:** Fernando Yue Cesena, Giuliano Generoso, Itamar de S. Santos, Alexandre C. Pereira, Marcio S. Bittencourt, Raul D. Santos, Paulo A. Lotufo, Isabela M. Benseñor

**Affiliations:** 1 Brazil Instituto Dante Pazzanese de Cardiologia, São Paulo, SP – Brazil; 2 Brazil Centro de Pesquisa Clínica e Epidemiológica, Hospital Universitário, Universidade de São Paulo, São Paulo, SP – Brazil; 3 USA Genetics Department, Harvard Medical School, Boston, Massachusetts – USA; 4 USA Heart and Vascular Institute, University of Pittsburgh Medical Center, Pittsburgh, Pennsylvania – USA; 5 Brazil Instituto do Coração (InCor), Hospital das Clínicas da Faculdade de Medicina da Universidade de São Paulo, São Paulo, SP – Brazil; 6 Brazil Hospital Israelita Albert Einstein, São Paulo, SP – Brazil

**Keywords:** Cardiovascular Diseases, Heart Disease Risk Factors, Body Mass Index, Risk Assessment, World Health Organization

## Introduction

In 2019, the World Health Organization (WHO) published revised charts for estimating the 10-year risk of cardiovascular disease (CVD).^1^ Two models are proposed: one based on laboratory tests including plasma total cholesterol and presence or absence of diabetes mellitus as predictors, and another based on the body mass index (BMI). In low-resource and office settings, when cholesterol levels and information on diabetes are not available, the BMI-based score can be used.^2^

In a previous study, we determined laboratory-based percentiles of the WHO CVD risk distribution, according to sex and age, in the Brazilian Longitudinal Study of Adult Health (ELSA-Brasil) baseline population.^3^ These percentiles were associated with the calculated risk of atherosclerotic CVD up to 75 years, regardless of the estimated 10-year risk. Expressing the risk percentile has been proposed to increase risk awareness and improve adherence to preventive measures.^3-5^

In this study, we sought to (1) determine sex- and age-specific percentiles of the distribution of the WHO non-laboratory-based CVD risk in the Brazilian population and (2) evaluate the agreement between WHO laboratory- and non-laboratory-based CVD risk scores.

## Methods

This study is a cross-sectional analysis of ELSA-Brasil baseline data collected from 2008 to 2010. ELSA-Brasil is a prospective cohort of 15,105 racially mixed employees from public universities and research institutions in six Brazilian cities.^6,7^ We included participants from 40 to 74 years old and excluded those with previous myocardial infarction, stroke, or revascularization procedures. The protocol for ELSA-Brasil was approved by the ethics committee of each participating institution, and all participants provided written informed consent.

Hypertension was considered in the presence of systolic blood pressure ≥140 mmHg, diastolic blood pressure ≥90 mmHg, or the use of medication to control blood pressure. Diabetes mellitus was defined as a self-reported diagnosis, use of specific medication, fasting blood glucose ≥126 mg/dL, glycated hemoglobin ≥6.5%, or a 2-hour blood glucose ≥200 mg/dL after 75 g load on oral glucose tolerance test. Detailed definitions of other variables reported in this study can be found elsewhere.^3^ Plasma total cholesterol levels and BMI were categorized according to the groups used for the WHO CVD risk calculation.^1,2^

Ten-year risk of experiencing a first fatal or nonfatal CVD event (related to coronary heart disease or stroke) was computed using the 2019 Updated WHO CVD risk charts calibrated for Tropical Latin America.^1,2^

To establish percentiles of the WHO non-laboratory-based CVD risk score distribution, we first sorted all possible values of the calculated risk within each age group. Then, we determined the distribution percentile corresponding to each score. Separate analyses were performed for each sex.

The sign test was applied to compare risk scores once differences between paired observations were not symmetric. A p-value < 0.05 was considered statistically significant. Agreement between risk values was assessed using Bland-Altman diagrams. All analyses were performed using R software and Microsoft Excel. The Shiny R package was used to develop a web application for calculating the 10-year CVD risk and the corresponding percentile for sex and age.

## Results

The study population (n = 13,366) was characterized by a higher female presence (55%) and a median age (interquartile range [IQR]) of 52 (46, 59) years (Supplemental Figure 1, Supplemental Table 1).

Supplemental Figure 2 shows the predicted 10-year CVD risk distribution according to sex and age group, while Supplemental Tables 2 to 8 report the percentiles of such distribution. These percentiles enabled the construction of risk-versus-percentile plots according to sex and age group (Figure 1).

**Figure 1:** – Percentiles of the predicted 10-year cardiovascular disease (CVD) risk distribution, according to sex and age group. The CVD risk was calculated using the non-laboratory-based risk score from the World Health Organization.

Overall, the BMI-based risk was slightly lower than the risk based on laboratory exams (median [IQR] 3% [2%, 5%] versus 4% [2%, 6%], respectively, p <0.001). Figure 2 shows the agreement between laboratory- and non-laboratory-based risk scores in females and males. The scores agreed in most participants (7,884 [59%]). The difference between the values (laboratory-based risk minus non-laboratory-based risk) ranged from -3% to 7% in females and from -5% to 17% in males.

**Figure 2:** – Bland-Altman diagrams for the agreement between WHO laboratory- and non-laboratory-based CVD risk scores according to sex. The blue line represents the mean of all differences (laboratory- minus non-laboratory-based scores), while the red and green lines represent the lower and the upper 95% limits of agreement, respectively. CVD: cardiovascular disease.

Supplemental Figures 3 to 5 depict the agreement between laboratory- and non-laboratory-based risk scores according to subgroups of interest. Among individuals without diabetes mellitus, the values were the same in 7,873 (70.5%), whereas the laboratory-based risk was numerically higher than the BMI-based risk in 2,176 (99.5%) participants with diabetes. The higher the total cholesterol level and the lower the BMI, the greater the tendency for a higher laboratory-based risk compared to non-laboratory-based risk.

A web application for calculating CVD risks and percentiles by sex and age can be accessed at https://bit.ly/3sGsIgK. An R code for creating new variables for WHO laboratory- and BMI-based CVD risks and the corresponding percentiles for sex and age in a dataset is available at https://bit.ly/3Pov250.

## Discussion

BMI-based CVD risk assessment serves as an option when cholesterol level and glycemic exams are unavailable^2^ and may offer community members a simpler method to calculate their own risk. Our findings indicate that BMI-based risk generally agrees with laboratory-based risk, but may be substantially lower in the presence of diabetes mellitus and very high total cholesterol. It is feasible to hypothesize that the non-laboratory-based risk score underestimates actual risk in these high-risk subgroups. However, definitive conclusions cannot be drawn once score performance metrics were not evaluated. We also observed that the BMI-based risk tends to be lower than the laboratory-based score as the BMI decreases. The question of which score is more accurate among individuals with low BMI remains unanswered.

Limitations of this study include the fact that the study population is not representative of the Brazilian population, with a higher female proportion, more white and less brown people compared to the 2022 Brazilian Census.^8^ Moreover, this agreement analysis may not be applicable to other populations with different demographic characteristics. Finally, it must be stressed that risk calculation represents only the first step in CVD risk stratification, and clinical practice should also consider several other factors and evaluation of subclinical atherosclerosis.^9^

In conclusion, this study determined percentiles of the WHO non-laboratory-based CVD risk distribution by sex and age in the Brazilian population. Compared to the laboratory-based strategy, the non-laboratory approach leads to the same risk score in most individuals, but tends to underestimate the risk among males, individuals with diabetes, higher LDL-c, or lower BMI.
